# Association Between Dietary Quality and Pulmonary Function Among Older Adults: Results from the Korea National Health and Nutrition Examination Survey, 2024

**DOI:** 10.3390/nu18213467

**Published:** 2026-10-22

**Authors:** Seong-Uk Baek, Jin-Ha Yoon

**Affiliations:** 1 Graduate School, Yonsei University College of Medicine, Seoul 03722, Republic of Korea; 2 Department of Preventive Medicine, Yonsei University College of Medicine, Seoul 03722, Republic of Korea; 3 Institute for Innovation in Digital Healthcare, Yonsei University Health System, Seoul 03722, Republic of Korea; 4 The Institute for Occupational Health, Yonsei University College of Medicine, Seoul 03722, Republic of Korea

**Keywords:** pulmonary function test, dietary pattern, respiratory health

## Abstract

Background: Dietary quality is important for preventing and managing chronic diseases in older adults; however, its association with respiratory health remains insufficiently understood. This cross-sectional study examined the association between dietary quality and lung function among older adults in South Korea. Methods: This cross-sectional study included 1021 adults aged ≥65 years from the 2024 Korea National Health and Nutrition Examination Survey. Dietary quality was measured using the Korean Healthy Eating Index (KHEI; range, 0–100). The forced expiratory volume in 1s (FEV1), forced vital capacity (FVC), FEV1/FVC ratio, forced mid-expiratory flow between 25% and 75% of FVC (FEF25–75%), and peak expiratory flow (PEF) were measured using spirometry. Linear regression models were used with beta coefficients and 95% confidence intervals (CIs). Results: The mean (standard deviation [SD]) KHEI score was 67.41 (10.97). The mean (SD) values for the lung function parameters were 2.95 (0.72) L for FVC, 2.21 (0.54) L for FEV1, 75.22 (7.68)% for the FEV1/FVC ratio, 1.93 (0.79) L/s for FEF25–75%, and 6.03 (1.73) L/s for PEF. The β (95% CI) for the relationship between the KHEI (unit = 1-SD increment) and lung function outcomes was 0.013 (−0.016–0.041) L for FVC, 0.028 (0.003–0.053) L for FEV1, 0.539 (0.129–0.950) % for the FEV1/FVC ratio, 0.057 (0.010–0.104) L/s for FEF25–75%, and 0.066 (−0.018–0.149) L/s for PEF. Conclusions: KHEI was positively associated with FEV1, FEV1/FVC, and FEF25–75%, whereas no clear associations were observed with FVC or PEF.

## 1. Introduction

Maintaining optimal lung function is crucial for promoting well-being in older adults. Chronic respiratory diseases, such as chronic obstructive pulmonary disease (COPD), asthma, or interstitial lung disease, impose a significant global health burden, particularly among older individuals [1,2]. Physiological and structural changes related to aging, such as reduced alveolar elasticity or respiratory muscle function, contribute to a decline in lung function in older adults [3,4]. Therefore, improving lung function in older adults is of utmost interest to promote healthy aging.

Dietary quality is essential for preventing chronic diseases, promoting wellbeing, and maintaining physical and functional capacity in older adults [5]. High dietary quality acts as a protective factor against cardiovascular diseases and all-cause mortality in older adults [6,7,8]. A recent study in South Korea using the Korean Healthy Eating Index (KHEI) indicated that older adults showed declining trends in the consumption of fresh fruits and vegetables, whole grains, and fiber, whereas fat consumption increased [9]. Such trends are attributable to the growing prevalence of Western-style eating habits alongside traditional Korean dietary practices, as well as the growing popularity of ultra-processed foods [10].

Dietary quality has emerged as an important lifestyle strategy for maintaining lung function and preventing pulmonary diseases. Previous studies have linked various dietary quality indices to lung function. For instance, the carbohydrate-to-fiber intake ratio was negatively associated with lung function parameters such as forced vital capacity (FVC), forced expiratory volume in 1s (FEV1), and peak expiratory flow (PEF) among adults [11]. A cohort study revealed that high dietary quality was associated with a slower decline in FEV1, FVC, and FEV1/FVC ratio among patients with COPD [12]. Additionally, dietary quality measures, including the dietary inflammatory index, mini nutritional assessment score, and Mediterranean diet score, have been associated with FEV1, FVC, FEV1/FVC ratio, and PEF in older adults [13,14,15]. These dietary quality measures generally reflect an adequate intake of fruits, vegetables, and protein, along with a moderate intake of fat and added sugars. The precise mechanisms linking dietary quality and lung function are yet to be fully understood; individuals with higher dietary quality tend to consume foods rich in antioxidants and proteins, which may be associated with lower systemic inflammation and better muscle health, potentially contributing to better lung function [16].

Despite growing evidence of an association between dietary quality and lung function, most studies have been conducted in the US or other Western populations whose dietary practices may differ considerably from those of South Korea and other Asian populations [11,12,17]. Evidence regarding the association between dietary quality and lung function among older adults in South Korea remains limited. Unlike previously used dietary indices, the KHEI is a validated diet quality index developed to quantitatively assess adherence to Korean dietary patterns based on Korean food culture and the Ministry of Health and Welfare’s Dietary Guidelines for Koreans [18]. Therefore, examining the association between KHEI and lung function may provide novel insights into the relationship between adherence to a balanced Korean-style diet and pulmonary function. Therefore, this study examined the association between dietary quality and lung function in older adults in South Korea using a nationally representative dataset.

## 2. Methods

### 2.1. Sample

Our cross-sectional analysis comprised individuals from the Korea National Health and Nutrition Examination Survey (KNHANES) by the Korea Disease Control and Prevention Agency (KDCA). Annually, the KNHANES recruits a nationally representative sample of people dwelling in South Korea using a multistage clustered probability sampling method, in which 192 enumeration districts covering the nation serve as primary sampling units [19]. This study included individuals from the 2024 cycle of the KNHANES, during which lung function testing resumed following the COVID-19 pandemic.

**Figure 1:**
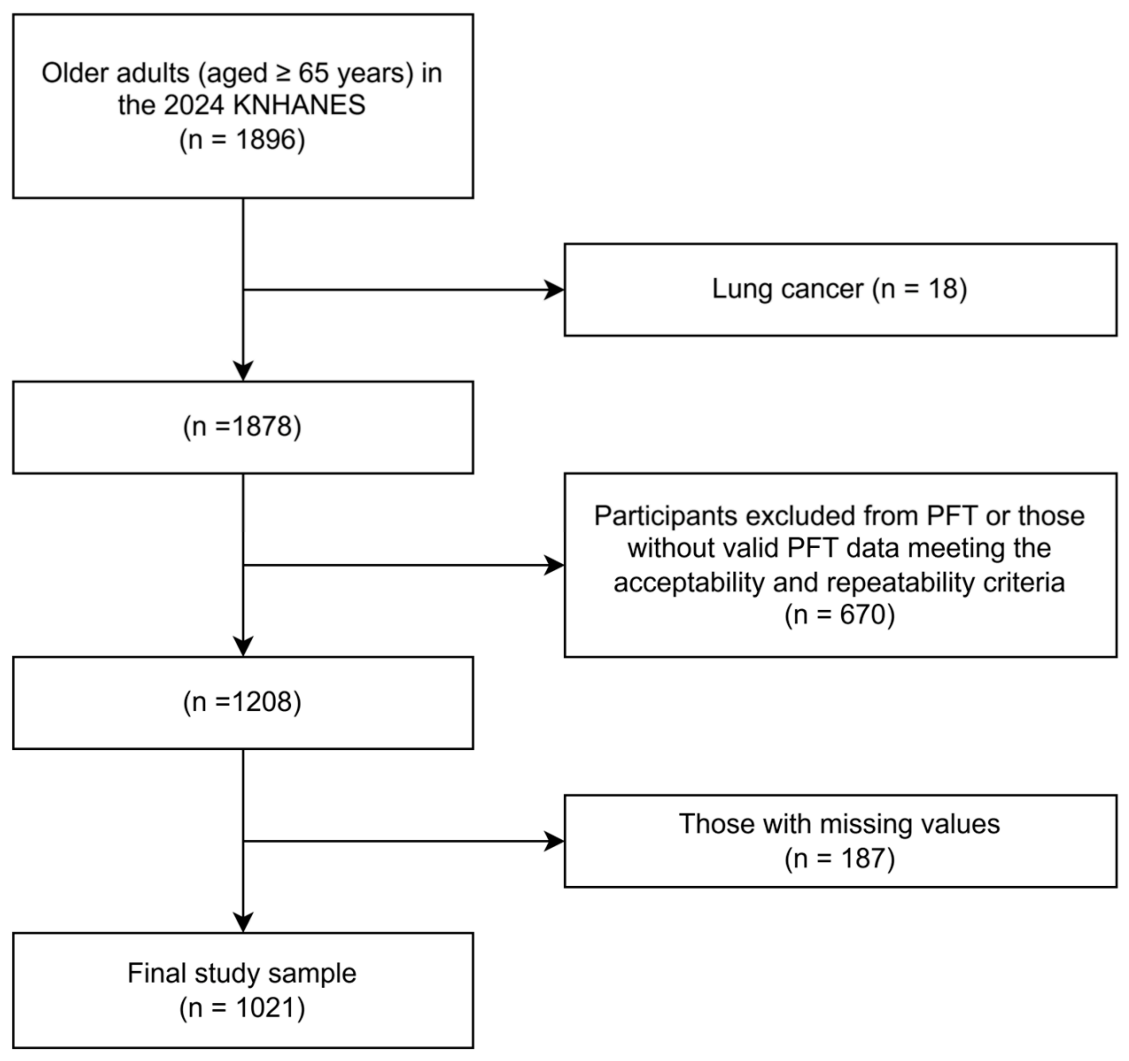
Flowchart of the study sample selection. (KNHANES, Korea National Health and Nutrition Examination Survey; PFT, pulmonary function test).

Figure 1 illustrates the sample-selection procedure. The initial sample comprised 1896 adults aged ≥65 years who participated in the 2024 KNHANES, which included a nutritional survey. Firstly, individuals with a history of lung cancer were excluded (n = 18). Subsequently, patients without valid lung-function data (n = 670) were excluded. After excluding individuals with missing data (n = 187), the final study sample comprised 1021 older adults. The Institutional Review Board of the KDCA granted ethical approval (2022-11-16-R-03). All the participants provided written informed consent.

### 2.2. Dietary Quality

Dietary quality was measured using the KHEI, which has good reliability and validity in the Korean population [20]. Developed after the Healthy Eating Index in the US [21], the KHEI includes 14 items assessing adequate intake of foods such as vegetables, fruits, protein-rich foods, and grains; moderate intake of saturated fatty acids, sodium, and added sugars; balance in energy derived from carbohydrates and fat; and adequate total energy intake. Scores range from 0 to 100, with a higher score representing better overall dietary quality. Dietary intake was assessed using a single-day 24-h dietary recall administered by trained dietitians to enhance the accuracy and validity of the nutritional assessment. Details regarding the specific KHEI items and scoring system have been previously described [20] and are presented in Table S1.

### 2.3. Lung Function Test

Trained technicians performed lung function tests using a Vyntus Spiro spirometer (CareFusion, San Diego, CA, USA). All procedures were performed in accordance with the standardization guidelines established by the American Thoracic Society and European Respiratory Society for calibration and quality control, and the Korean guidelines for pulmonary function testing [22,23]. For quality control, technicians underwent regular training and assessments twice a year, and the pulmonary function testing equipment was inspected twice annually and maintained on an ongoing basis. For safety reasons, individuals with contraindications to spirometry, including ophthalmic surgery, recent surgery, cardiovascular events, hemoptysis, tuberculosis, chest or abdominal pain, systolic blood pressure >200 mmHg, diastolic blood pressure >140 mmHg, urinary incontinence, or cognitive impairment such as severe dementia, were excluded from spirometry. All spirometry measurements were performed before bronchodilator administration. Spirometry tests that met the criteria for acceptability and reproducibility were retained for the analysis. To meet the acceptability criteria, spirometry maneuvers had to be free of the following: (1) an improper start of expiration, (2) coughing during the maneuver, (3) premature termination of expiration, (4) a Valsalva maneuver or interruption of airflow during the test, (5) air leakage, (6) obstruction of the mouthpiece, and (7) insufficient effort. Reproducibility criteria were considered satisfied when the difference between the two largest FVC values was ≤150 mL and the difference between the two largest FEV1 values was ≤150 mL [24]. Spirometry was performed with a minimum of two and a maximum of eight maneuvers. A maneuver was considered acceptable when the volume–time curve reached a plateau during expiration within 6s. Reproducibility was considered satisfactory when the difference between the highest and second-highest values of FVC and FEV1 among at least two acceptable maneuvers was <150 mL. Participants excluded due to contraindications and those with unacceptable or non-reproducible PFT maneuvers were both recorded as having missing PFT values. Five pulmonary function parameters were evaluated: FVC, FEV1, FEV1/FVC ratio, forced mid-expiratory flow between 25% and 75% of forced vital capacity (FEF25–75%), and PEF. In addition to the major lung function parameters, including FVC, FEV1, and the FEV1/FVC ratio, FEF25–75% and PEF were also included as outcomes to assess whether the associations between dietary quality and lung function were consistent across different parameters. Prebronchodilator PFT values, including the FEV1/FVC ratio, were used to evaluate lung function under baseline conditions, reflecting respiratory function and airflow limitation before bronchodilator administration. These values may be lower than postbronchodilator measurements. This approach also facilitates comparisons with the general population, in whom postbronchodilator measurements are not routinely conducted.

### 2.4. Control Variables

The following socio-demographic variables were adjusted for: sex, age (years), income level (Q1–Q4), education level (elementary school or lower, middle school, or high school or higher), marital status (married or unmarried), exercise (<150 min/week or ≥150 min/week), alcohol consumption (alcohol use disorder identification test—consumption, AUDIT-C), cumulative smoking exposure (non-exposed, <20 pack-years, and ≥20 pack-years), body mass index (kg/m^2^), height (m^2^), hypertension (no or yes), diabetes mellitus (no or yes), and ischemic heart disease (no or yes).

### 2.5. Data Analysis

The associations between the KHEI and lung function parameters were determined using linear regression models, using beta coefficients (β) and 95% confidence intervals (CIs). Both the crude and adjusted models were fitted. To explore the potential nonlinear relationship between the KHEI and lung function parameters, restricted cubic spline functions were employed using four knots [25]. As recommended in the previous literature [26], four knots at the 5th, 35th, 65th, and 95th percentiles were selected to provide sufficient flexibility while minimizing the potential for overfitting. Additionally, the restricted cubic spline model estimated marginal predicted values across the range of KHEI scores, and no specific reference value was prespecified because the results were presented as predicted values rather than relative measures. Data analysis was performed using the R software (version 4.6.1). The complex survey design was applied to the regression models using the R package “survey” [27]. Specifically, sampling weights (variable name: “wt_tot”), strata (“kstrata”), and primary sampling units (“psu”) were specified in the “svydesign” function [28]. Subsequently, survey-weighted linear regression models were fitted using the “svyglm” function based on the complex survey design object specified in the preceding step. For regression models, multicollinearity was assessed using the adjusted generalized variance inflation factor (GVIF), which is calculated using the R package “car” and its function “vif.” An adjusted GVIF value greater than 2 was considered indicative of multicollinearity. Additionally, to account for multiple comparisons across the five outcomes and control the potential for type I errors, *p*-values were adjusted using the Benjamini–Hochberg false discovery rate (FDR) procedure [29].

## 3. Results

The features of the samples are listed in Table 1. The sample comprised 49.86% women, with a mean age of 71.57 years. The mean (standard deviation [SD]) KHEI score was 67.41 (10.97). The mean (SD) values for the lung function parameters were 2.95 (0.72) L for FVC, 2.21 (0.54) L for FEV1, 75.22 (7.68)% for the FEV1/FVC ratio, 1.93 (0.79) L/s for FEF25–75%, and 6.03 (1.73) L/s for PEF. Compared with included participants, excluded participants (n = 875) were more likely to be female, older, have lower education levels, and have lower income levels (Table S2). They were also more likely to be unmarried, physically inactive, have lower AUDIT-C scores, have lower cumulative smoking exposure, and have shorter height. Additionally, excluded participants had lower KHEI scores and lower FVC and PEF values than included participants.

Table 2 shows the results of regression models for the relationship between dietary quality and lung function. In the adjusted models, the β (95% CI) for the association between the KHEI (unit = 1-SD increment) and lung function outcomes was 0.013 (−0.016–0.041) L for FVC, 0.028 (0.003–0.053) L for FEV1, 0.539 (0.129–0.950) % for the FEV1/FVC ratio, 0.057 (0.010–0.104) L/s for FEF25–75%, and 0.066 (−0.018–0.149) L/s for PEF. The FDR-corrected *p*-value for each pulmonary function parameter was 0.380 for FVC, 0.048 for FEV1, 0.043 for the FEV1/FVC ratio, 0.043 for FEF25–75%, and 0.154 for PEF. Across all regression models, the adjusted GVIF values were below 2 for all variables, indicating no evidence of multicollinearity.

**Figure 2:**
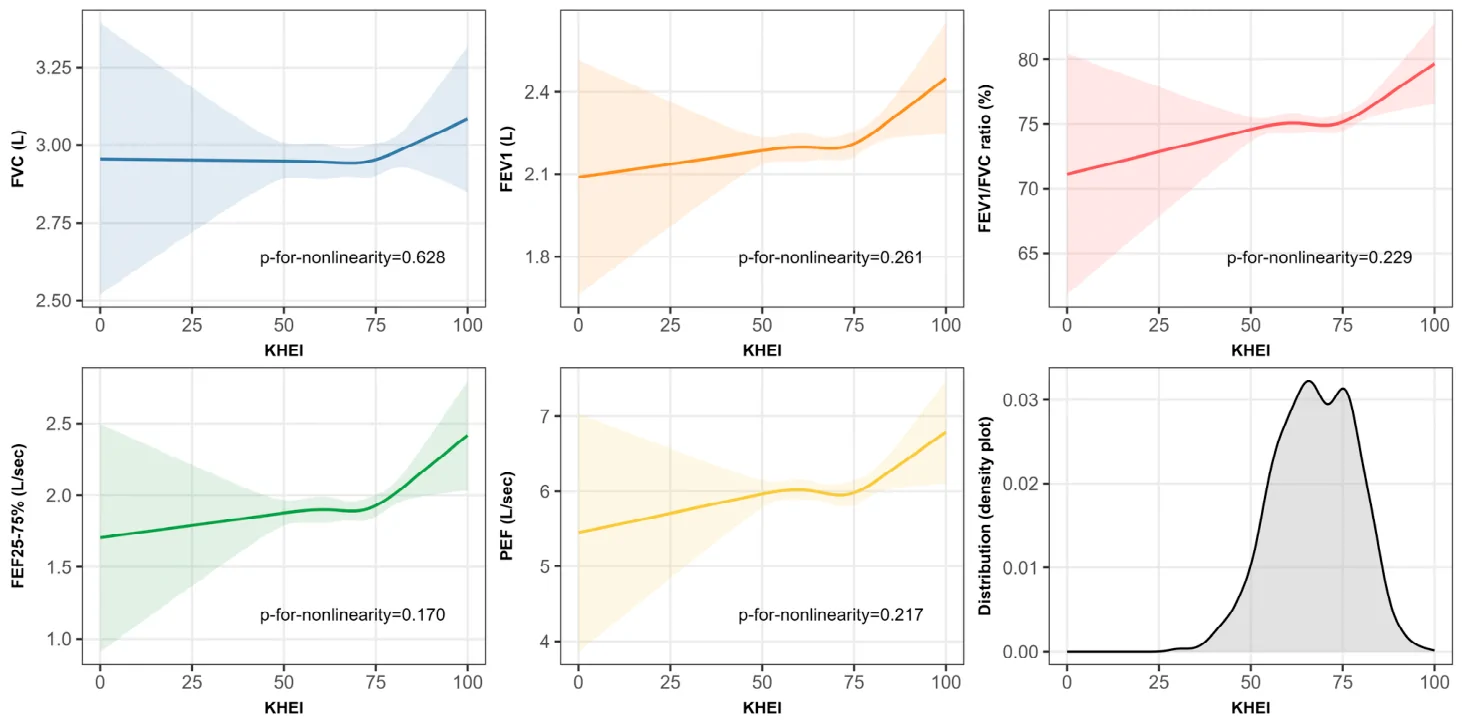
Nonlinear associations of dietary quality and lung function using restricted cubic spline models. All models controlled for sex, age, education, income, marital status, exercise, alcohol consumption, smoking history, body mass index, height, hypertension, diabetes mellitus, and history of ischemic heart disease as covariates. The plot in the lower-right corner shows the distribution of KHEI values in the sample using a density plot. (KHEI, Korean Healthy Eating Index; FEV1, forced expiratory volume in 1s; FVC, forced vital capacity; FEF25–75%, forced mid-expiratory flow between 25% and 75% of forced vital capacity; PEF, peak expiratory flow).

The nonlinear associations between dietary quality and lung function are shown in Figure 2. The *p* values for nonlinearity were all >0.05 across the lung function parameters, suggesting an overall linear association between KHEI and lung function outcomes. However, as shown in the density plot of KHEI values in the sample, observations with KHEI scores below 50 were sparse, resulting in wide confidence intervals at the lower end of the distribution. This limited sample size may have reduced statistical power to detect associations in this range, and the possibility of a local nonlinear pattern in the association between KHEI and pulmonary function parameters could not be completely ruled out.

## 4. Discussion

This study examined the relationship between KHEI and lung function in older adults in South Korea. The KHEI was positively associated with several lung function parameters, including FEV1, FEV1/FVC ratio, and FEF25–75%. However, no clear associations were observed between the KHEI and FVC and PEF. The findings suggest that dietary quality is positively associated with lung function in older adults in South Korea. However, longitudinal research should be conducted to elucidate the temporal relationship between dietary quality and lung function.

These findings align with those of prior studies showing a link between dietary quality or patterns and lung function among older adults. For example, a cross-sectional study of older Italian adults showed that poor dietary quality, as assessed by the dietary inflammatory index, is associated with low FEV1 and FVC [13]. A study of 50 older adults in South Korea showed that dietary quality measured using the Mini Nutritional Assessment was positively associated with FEV1 and FEV1/FVC ratio [14]. Additionally, a higher Mediterranean diet score was positively associated with the PEF among older adults in the US [15]. Regarding dietary quality measures, our findings are consistent with those of a previous study in the US, which revealed a positive association between HEI and the FEV1/FVC ratio [30]. Compared with a previous study conducted in South Korea using a relatively small sample size [14], our study used a nationwide sample of older adults. Additionally, to the best of our knowledge, this is the first study to examine the relationship between KHEI and lung function.

The effect sizes per one-SD increment in the KHEI were relatively modest, despite statistical significance. For instance, a previous study of community-dwelling older adults reported a mean decline of 0.043 L in FEV1 among those without COPD [31], whereas the β coefficient of 0.028 L observed in the present study was smaller. Therefore, statistical significance should not necessarily be interpreted as indicating clinical significance. Nevertheless, even modest associations may have population-level relevance. Additionally, compared with major lung function parameters such as FEV1, FVC, and the FEV1/FVC ratio, which are central to clinical assessment of lung function, FEF25–75% and PEF have less well-established clinical significance and greater variability [32]. These limitations should be considered when interpreting the findings, and associations with these parameters may not necessarily indicate clinical implications.

The association of dietary quality with lung function observed in this study may be attributable to several mechanisms. Better dietary quality may be associated with greater anti-inflammatory potential, which may help attenuate chronic low-grade systemic inflammation [33,34]. For instance, HEI is negatively associated with inflammatory biomarkers including *C*-reactive protein [35,36,37]. Additionally, favorable dietary quality is negatively associated with inflammatory biomarkers, such as the systemic inflammation response index and systemic immune-inflammation index [38]. High dietary quality, characterized by a sufficient intake of fruits, vegetables, or whole grains, may be associated with a greater intake of dietary antioxidants, including vitamins. Previous studies have shown that dietary antioxidants may help regulate inflammatory responses by scavenging reactive oxygen species and inhibiting NF-κB activation [39,40]. Chronic low-grade systemic inflammation is implicated in airway remodeling and mucus hypersecretion and may be associated with impaired lung function [41]. Therefore, dietary quality among older adults may be positively associated with lung function through mechanisms involved in the mitigation of systemic inflammation. Unlike FEV1 and the FEV1/FVC ratio, FVC and PEF were not significantly associated with the KHEI. Although the precise mechanisms underlying these differences across lung function parameters remain unclear, diet quality-related inflammatory processes may involve low-grade inflammation affecting the small airways, potentially contributing to the observed associations between the KHEI and lower FEV1 and FEV1/FVC ratio values. Nevertheless, this explanation does not fully account for the lack of a significant association with PEF. The relatively modest sample size may have limited statistical power to detect an association between the KHEI and lung function parameters. However, the biological mechanisms described above were not investigated in the present study and therefore remain hypothetical, representing potential explanations rather than mechanisms directly demonstrated by our findings.

This study had limitations. First, a causal relationship between dietary quality and lung function could not be determined because of the cross-sectional design of the study. For example, the possibility of reverse causation should be considered, as individuals with poor health conditions may have reduced access to or consumption of healthy foods. Therefore, the temporal relationship between dietary quality and lung function should be examined further in future longitudinal studies. Secondly, the influence of residual confounding factors should be considered. For instance, occupational or environmental exposure and comorbidities that may be associated with both diet quality and lung function [42,43,44] could not be accounted for in our analysis because information on these factors was unavailable in the dataset. Additionally, information on the use of medications for respiratory diseases, including corticosteroids and beta-agonists, was unavailable in the current dataset, precluding a more detailed analysis of respiratory conditions and medication use. This represents an important limitation of the present study. Third, although the KHEI is a validated measure of dietary quality among Korean adults, it was assessed using a 24-h dietary recall, which may not fully capture day-to-day variations in dietary intake. A single-day 24-h dietary recall may not fully capture usual dietary intake because of within-person variation, potentially leading to exposure misclassification. Additionally, a single 24-h dietary recall may not adequately capture usual dietary intake over the long term, potentially introducing random measurement error in the assessment of dietary quality [45,46]. Despite the nutritional assessment being guided by trained dietitians, the 24-h recall method remains susceptible to recall bias and social desirability bias. Future studies incorporating repeated or long-term nutritional assessments may improve the quality and accuracy of dietary measurements. Fourth, a substantial proportion of the initial participants were excluded from the analysis, raising concerns about potential selection bias. Excluded participants were more likely to be older, have lower socioeconomic status, and have lower FVC and PEF values than included participants. The exclusion of these individuals may have introduced systematic bias and limited the generalizability of the findings.

An important strength of this study is the use of the validated KHEI score to assess dietary quality. In addition, spirometry data were obtained through standardized procedures and strengthened the robustness of the study. Finally, this study is among the few to provide evidence of the association of dietary quality with lung function in older adults in South Korea.

## 5. Conclusions

The results showed that dietary quality is associated with lung function test results in older adults in South Korea. Higher KHEI scores were positively associated with several lung function parameters, including FEV1, FEV1/FVC ratio, and FEF25–75%, whereas no clear associations were observed with FVC or PEF. However, the possibility of reverse causation inherent in the cross-sectional design should be considered when interpreting our findings; nevertheless, improving dietary quality may be a potential target for promoting respiratory health among older adults. Therefore, our findings provide preliminary evidence of an association between dietary quality and lung function, and further prospective studies are needed to elucidate this longitudinal association.

## Figures and Tables

**Table 1:** Sample characteristics.

	N (%)	Mean (SD)
Sex		
Male	479 (50.24%)	
Female	542 (49.76%)	
Age (years)		71.57 (4.66)
Income level		
Lowest (Q1)	203 (18.39%)	
Low	264 (25.49%)	
High	263 (26.21%)	
Highest (Q4)	291 (29.91%)	
Education level		
Elementary school or lower	392 (36.27%)	
Middle school	237 (23.22%)	
High school or higher	392 (40.51%)	
Marital status		
Married	739 (73.88%)	
Unmarried	282 (26.12%)	
Exercise		
No	675 (65.05%)	
Yes	346 (34.95%)	
AUDIT-C		2.29 (3.11)
Cumulative smoking exposure		
Non-exposure	628 (59.47%)	
<20 pack-years	151 (15.62%)	
≥20 pack-years	242 (24.91%)	
Body mass index (kg/m^2^)		24.16 (3.05)
Height (cm)		160.31 (8.53)
Hypertension		
No	396 (40.52%)	
Yes	625 (59.48%)	
Diabetes mellitus		
No	721 (70.16%)	
Yes	300 (29.84%)	
Ischemic heart disease		
No	952 (92.98%)	
Yes	69 (7.02%)	
KHEI		67.41 (10.97)
FVC (L)		2.95 (0.72)
FEV1 (L)		2.21 (0.54)
FEV1/FVC ratio (%)		75.22 (7.68)
FEF25–75% (L/s)		1.93 (0.79)
PEF (L/s)		6.03 (1.73)

SD, standard deviation; KHEI, Korean Healthy Eating Index; FEV1, forced expiratory volume in 1 s; FVC, forced vital capacity; FEF25–75%, forced mid-expiratory flow between 25% and 75% of forced vital capacity; PEF, peak expiratory flow; AUDIT-C, alcohol use disorder identification test—consumption. Values were presented as unweighted n with survey-weighted % for categorical variables and survey-weighted mean with standard deviation for continuous variables.

**Table 2:** Associations between dietary quality and lung function using linear regression models.

	Crude Model		Adjusted Model	
	β (95% CI)	*p* Value	β (95% CI)	*p* Value
Lung function				
FVC (L)	0.024 (−0.021, 0.069)	0.299	0.013 (−0.016, 0.041)	0.380
FEV1 (L)	0.046 (0.011, 0.081)	0.010	0.028 (0.003, 0.053)	0.029
FEV1/FVC ratio (%)	0.878 (0.437, 1.319)	<0.001	0.539 (0.129, 0.950)	0.010
FEF25–75% (L/s)	0.092 (0.043, 0.140)	<0.001	0.057 (0.010, 0.104)	0.017
PEF (L/s)	0.106 (−0.009, 0.221)	0.071	0.066 (−0.018, 0.149)	0.123

FEV1, forced expiratory volume in 1s; FVC, forced vital capacity; FEF25–75%, forced mid-expiratory flow between 25% and 75% of forced vital capacity; PEF, peak expiratory flow; CI, confidence interval. The adjusted models included sex, age, education, income, marital status, exercise, alcohol consumption, smoking history, body mass index, height, hypertension, diabetes mellitus, and history of ischemic heart disease as covariates. The beta coefficients represent changes in the respective pulmonary function parameter per one-standard-deviation increase in the Korean Healthy Eating Index.

## Data Availability

Raw data is accessible at https://knhanes.kdca.go.kr/knhanes/rawDataDwnld/rawDataDwnld.do (accessed on 3 August 2026). Researchers can download datasets for individual survey years after providing their name, institutional affiliation, and email address.

## Supplementary Materials

The following supporting information can be downloaded at https://www.mdpi.com/article/10.3390/nu18213467/s1. Table S1. Survey items and scoring system of the Korean Healthy Eating Index; Table S2. Comparison of the characteristics between included and excluded participants. Values were presented as unweighted n with survey-weighted % for categorical variables and survey-weighted mean with standard deviation for continuous variables.
