# Supplementary material for: Association Between Dietary Quality and Pulmonary Function Among Older Adults: Results from the Korea National Health and Nutrition Examination Survey, 2024
Source: Nutrients. 2026 Oct 22;18(21):3467. doi: 10.3390/nu18213467 (PMC13765497; doi:10.3390/nu18213467)
Supplement: Supplementary file 1 [file nutrients-18-03467-s001.zip › nutrients-4609265-supplementary.pdf]

Table S1. Survey items and scoring system of the Korean Healthy Eating Index

	Scoring		
	Score	Criteria	
		Minimum score	Maximum score
**Total KHEI score**	0–100		
**Adequacy component**			
**Total adequacy score**			
Have breakfast	0–10	0 times/week	≥5 times/week
Mixed grains intake	0–5	0 serving/day	≥0.3 serving/day
Total fruits intake	0–5	0 serving/day	≥2/1 (men/women) serving/day
Fresh fruits intake	0–5	0 serving/day	≥1/0.5 (men/women) serving/day
Total vegetables intake	0–5	0 serving/day	≥8/6 (men/women) serving/day
Vegetables intake excluding Kimchi and pickled vegetables intake	0–5	0 serving/day	≥5/3 (men/women) serving/day
Meat, fish, eggs, and beans intake	0–10	0 serving/day	≥4/2.5 (men/women) serving/day
Milk and milk products intake	0–10	0 serving/day	≥1 serving/day
**Moderation component**			
**Total moderation score**			
Percentage of energy from saturated fatty acid	0–10	>10% of total energy intake	<7% of total energy intake
Sodium intake	0–10	>6,500 mg/day	≤2,000 mg/day
Percentage of energy from sugars	0–10	>20% of total energy intake	≤10% of total energy intake
**Energy balance component**			
**Total energy balance score**			
Percentage of energy intake from carbohydrate	0–5	<50% or >75% of total energy intake	55–65% of total energy intake
Percentage of energy intake from fat	0–5	<10% or >35% of total energy intake	15–30% of total energy intake
Energy intake	0–5	<60% or >140% of the EER	75–125% of the EER

Korean Healthy Eating Index, Korean Healthy Eating Index; EER, estimated energy requirement
Detailed information can be found at the previous literature.[1]

[1] Yun S, Park S, Yook SM, Kim K, Shim JE, Hwang JY, Oh K. Development of the Korean Healthy Eating Index for adults, based on the Korea National Health and Nutrition Examination Survey. Nutr Res Pract. 2022 Apr;16(2):233-247. doi: 10.4162/nrp.2022.16.2.233.

Table S2 Comparison of the characteristics between included and excluded participants. Values were presented as unweighted n with survey-weighted % for categorical variables and survey-weighted mean with standard deviation for continuous variables.

	**Included sample N = 1021**	**Excluded sample N = 875**	**P value ***
	**N (%) or Mean (SD)**	**N (%) or Mean (SD)**	
**Sex**			<0.001
Male	479 (50.2%)	310 (39.4%)	
Female	542 (49.8%)	565 (60.6%)	
**Age (years)**	71.6 (4.7)	74.5 (5.2)	<0.001
**Income level**			<0.001
Lowest (Q1)	203 (18.4%)	264 (28.8%)	
Low	264 (25.5%)	204 (22.4%)	
High	263 (26.2%)	213 (25.4%)	
Highest (Q4)	291 (29.9%)	182 (23.4%)	
Number of missing values	12		
**Education level**			<0.001
Elementary school or lower	392 (36.3%)	433 (53.7%)	
Middle school	237 (23.2%)	125 (16.0%)	
High school or higher	392 (40.5%)	216 (30.3%)	
Number of missing values	101		
**Marital status**			<0.001
Married	739 (73.9%)	512 (61.0%)	
Unmarried	282 (26.1%)	358 (39.0%)	
Number of missing values	5		
**Exercise**			0.012
No	675 (66.1%)	512 (74.3%)	
Yes	346 (33.9%)	177 (25.7%)	
Number of missing values	186		
**AUDIT-C**	2.3 (3.1)	1.5 (2.4)	<0.001
Number of missing values	97		
**Cumulative smoking exposure**			<0.001
Non-exposure	628 (59.5%)	564 (69.4%)	
< 20 pack-years	151 (15.6%)	96 (14.2%)	
≥ 20 pack-years	242 (24.9%)	113 (16.3%)	
Number of missing values	102		
**Body mass index (kg/m^2)**	24.2 (3.0)	23.9 (3.4)	0.231
Number of missing values	73		
**Height (cm)**	160.3 (8.5)	157.8 (9.0)	<0.001
Number of missing values	73		
**Hypertension**			0.183
No	396 (40.5%)	322 (37.4%)	
Yes	625 (59.5%)	538 (62.6%)	
Number of missing values	15		
**Diabetes mellitus**			0.569
No	721 (70.2%)	502 (68.7%)	
Yes	300 (29.8%)	225 (31.3%)	
Number of missing values	148		
**Ischemic heart disease**			0.054
No	952 (93.0%)	631 (90.3%)	
Yes	69 (7.0%)	66 (9.7%)	
Number of missing values	178		
**KHEI**	67.4 (11.0)	65.3 (12.2)	<0.001

Number of missing values	1		
**FVC (L)**	3.0 (0.7)	2.8 (0.7)	0.034
Number of missing values	680		
**FEV1 (L)**	2.2 (0.5)	2.1 (0.6)	0.117
Number of missing values	680		
**FEV1/FVC ratio (%)**	75.2 (7.7)	75.8 (8.8)	0.429
Number of missing values	697		
**FEF25–75% (L/sec)**	1.9 (0.8)	2.0 (0.9)	0.748
Number of missing values	680		
**PEF (L/sec)**	6.0 (1.7)	5.6 (1.7)	0.010
Number of missing values	680		

* Survey-weighted chi-squared test for categorical variables and survey weighted t-test for continuous variables.
